# Metabolic Activity and mRNA Levels of Human Cardiac CYP450s Involved in Drug Metabolism

**DOI:** 10.1371/journal.pone.0015666

**Published:** 2010-12-14

**Authors:** Veronique Michaud, Martin Frappier, Marie-Christine Dumas, Jacques Turgeon

**Affiliations:** Research Centre of the University of Montreal Hospital Centre (CRCHUM), Centre Hospitalier de l'Université de Montréal and Faculty of Pharmacy, Université de Montréal, Montreal, Quebec, Canada; Dr. Margarete Fischer-Bosch Institute of Clinical Pharmacology, Germany

## Abstract

**Background:**

Tissue-specific expression of CYP450s can regulate the intracellular concentration of drugs and explain inter-subject variability in drug action. The overall objective of our study was to determine in a large cohort of samples, mRNA levels and CYP450 activity expressed in the human heart.

**Methodology:**

CYP450 mRNA levels were determined by RTPCR in left ventricular samples (n = 68) of explanted hearts from patients with end-stage heart failure. Samples were obtained from ischemic and non-ischemic hearts. In some instances (n = 7), samples were available from both the left and right ventricles. A technique for the preparation of microsomes from human heart tissue was developed and CYP450-dependent activity was determined using verapamil enantiomers as probe-drug substrates.

**Principal Findings:**

Our results show that CYP2J2 mRNA was the most abundant isoform in all human heart left ventricular samples tested. Other CYP450 mRNAs of importance were CYP4A11, CYP2E1, CYP1A1 and CYP2C8 mRNAs while CYP2B6 and CYP2C9 mRNAs were present at low levels in only some of the hearts analyzed. CYP450 mRNAs did not differ between ischemic and non-ischemic hearts and appeared to be present at similar levels in the left and right ventricles. Incubation of verapamil with heart microsomes led to the formation of nine CYP450-dependent metabolites: a major finding was the observation that stereoselectivity was reversed compared to human liver microsomes, in which the R-enantiomer is metabolized to a greater extent.

**Conclusions:**

This study determined cardiac mRNA levels of various CYP450 isozymes involved in drug metabolism and demonstrated the prevalent expression of CYP2J2 mRNA. It revealed that cardiomyocytes can efficiently metabolize drugs and that cardiac CYP450s are highly relevant with regard to clearance of drugs in the heart. Our results support the claim that drug metabolism in the vicinity of a drug effector site can modulate drug effects.

## Introduction

The cytochrome P450 (CYP450) is a superfamily of hemoproteins that are the terminal oxidases of the mixed function oxidase system involved in the biotransformation of endogenous compounds and xenobiotics.[1] To date, 57 genes and 58 pseudogenes of the CYP450 superfamily have been characterized from the human genome. (http://drnelson.utmem.edu/CytochromeP450.htlm) This superfamily is composed of 18 families of genes, four of which—CYP1, CYP2, CYP3 and CYP4—are involved in the metabolism of clinically used drugs.

Although CYP450 enzymes are mainly expressed in the liver, most extrahepatic tissues express CYP450 isozymes to varying degrees. The heart is no exception. Indeed, recent reports have demonstrated the presence of *CYP450* gene products in a cardiomyoblast cell line [2], in cultured rat cardiomyocytes [3], in the heart of several animal species such as rat [4], [5], rabbit [6], fish [7], sheep [8] and pig [9] and more importantly, in samples of a few explanted human hearts [10], [11], [12]. However, only a handful of studies have reported data on the relative expression of CYP450s from the CYP1-4 families in human heart tissues. When information is available, it is based on a small number (often <10) of explanted hearts from subjects with various conditions.[10], [11], [12], [13] For instance, Thum and Borlak report the presence of CYP1A1, CYP2B6, CYP2C8, CYP2C19, CYP2D6 and CYP4B1 mRNAs in seven explanted hearts. No mRNA for CYP3As could be detected in these samples.[10]

The functional role of CYP450s in cardiovascular health and disease has gained great interest in the scientific community.[14], [15] For example, several studies have shown the role of CYP450s expressed in cardiovascular tissues for the catabolism of endogenous compounds such as arachidonic acid and steroids.[16], [17] Arachidonic acid is transformed into epoxyeicosatrenoic acid metabolites (EETs) that regulate vascular tone and possess anti-inflammatory and anti-fibrotic properties.[15], [18] CYP450-mediated formation of estradiol and hydroxyestradiol is associated with a decrease in cardiomyocyte apoptosis and a protective role against cardiac hypertrophy.[19], [20] Furthermore, testosterone metabolism appeared to be increased in hypertrophied hearts due to the induction of CYP450s leading to increased levels of lipid peroxidation.[21]

Attention has been paid to the role of CYP2Cs in cardiovascular homeostasis. In particular, CYP2C9 may play a role in the onset and progression of cardiovascular diseases and in inflammatory processes since this isozyme can produce vasoreactive EETs and generate reactive oxygen species. Inhibition of CYP2C9 activity increased post-ischemic endothelium-dependant vasodilatation and reduced post-ischemic vascular superoxide production.[22] The infarct size was reduced and post-ischemic coronary flow was increased in rat hearts.[23] Fischer et al. report that flow-mediated dilation in human conductance arteries is reduced after infusion of sulfaphenazole, a CYP2C9 inhibitor, supporting the idea that CYP2C-dependent metabolites play a role in endothelium-mediated vasodilation.[24] They also observe that CYP-dependent flow-mediated dilation is preserved in patients with heart failure.[24] A relationship between CYP2J2, endogenous generation of EETs and cardioprotective effects has also been reported. Increased expression of CYP2J2 in mice hearts decreases ischemia-reperfusion damage through production of EETs which affect the K_ATP_ channel activity and MAP kinase signalling.[25]

Several drugs exert their pharmacological effects on the heart and are prone to being metabolized by CYP450 isozymes expressed in cardiac tissues. The cellular concentration of drugs within the heart may therefore be modulated by the intrinsic activity of CYP450 isozymes found in cardiac tissues. To date, little is known about the magnitude of drug metabolism occurring in the human heart and no information is available concerning site-specific drug-drug interactions as they pertain specifically to the heart.

In this light, the objectives of our study were: 1) to determine the relative levels of CYP450 mRNAs (CYP1, CYP2, CYP3 and CYP4 families) in a large cohort (n = 68) of explanted left ventricular heart samples from patients with end-stage heart failure undergoing cardiac transplant; 2) to compare the relative CYP1-4 mRNA levels in samples from ischemic and non-ischemic hearts; 3) to compare the relative CYP1-4 mRNA levels in left and right ventricular tissues in selected subjects (n = 7); 4) to demonstrate by immunohistochemical studies the presence of some CYP450s in human cardiac myocytes; 5) to develop procedures for the preparation of microsomes from human heart tissue; 6) to establish the CYP450-dependent activity of microsomes prepared from human heart ventricles using verapamil as a probe-substrate; and 7) to characterize the enantioselective metabolism of R- and S-verapamil in human heart microsomes. Verapamil was used as a probe-drug since it is known to be metabolized by several CYP450 isozymes, it possesses cardiovascular actions for which variability in CYP450 activities may be relevant. and finally, Walles et al. reported the formation of various verapamil metabolites in human heart samples in a pilot study.[26]

## Results

### CYP450 mRNA levels in human hearts

The demographic data and characteristics of patients and subjects from which samples were collected are summarised in Table 1. Our cohort was composed of 75 different heart samples, including 68 samples from the left ventricles of all subjects included in this study and 7 additional samples from the right ventricles of a subgroup of patients. All results presented in this section are expressed relative to the housekeeping gene *GAPDH*, uniformly expressed in all tissues analyzed.

**Table 1:** Characteristics of patients from whom samples were obtained.

Demographic data	
Gender (male:female)*	41∶23
Weight (kg)*	73±19
Height (cm)*	170±9
Number of left ventricle samplesNumber of right ventricle samples	687
Number of ischemic vs. non-ischemic hearts*	24∶40

*data are missing for 4 patients.

Mean ±*S.D.*

#### Relative CYP450 mRNA levels of measured in human hearts

**Figure 1:**
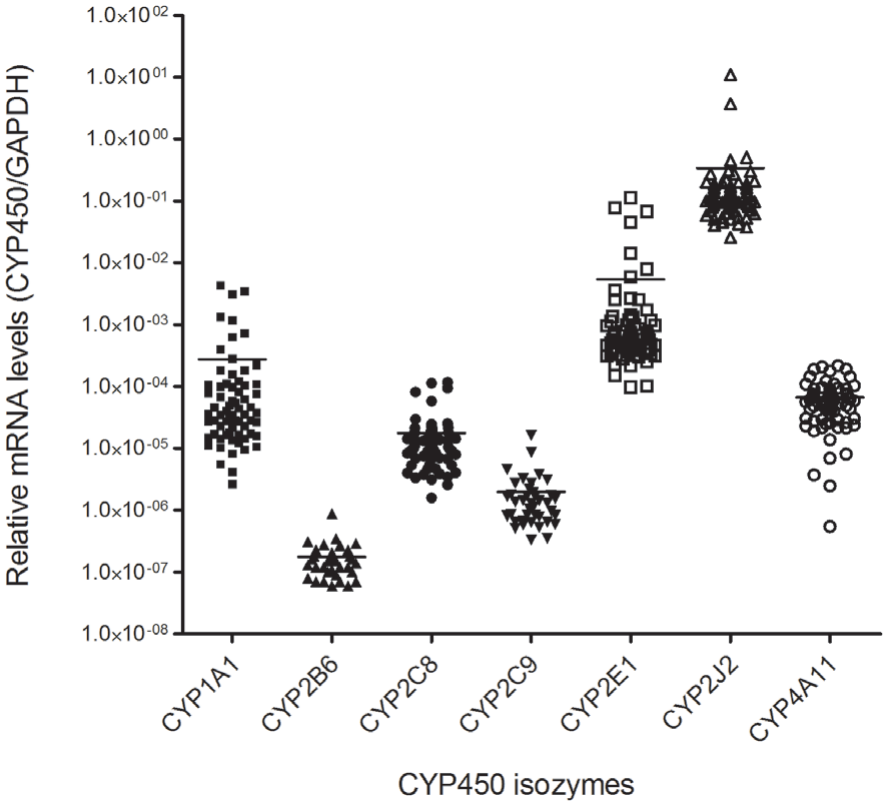
CYP450s mRNA levels in failing human hearts. Relative levels of CYP450 mRNAs measured in samples from human left ventricles. Levels were determined and compared using the 2^−ΔCT^ method without a calibrator.

Figure 1 illustrates mRNA levels of CYP450s of interest measured in the entire cohort of hearts studied. CYP2J2 mRNA levels largely exceeded (∼3 million to 62 times) those determined on average for other isozymes (p<0.008). CYP4A11, CYP2E1, CYP1A1 and CYP2C8 mRNAs were present at an intermediate level while CYP2B6 and CYP2C9 mRNAs were present at very low levels in some of the analyzed hearts.

#### Comparison of the relative CYP450 mRNA levels in ischemic and non-ischemic samples

**Figure 2:**
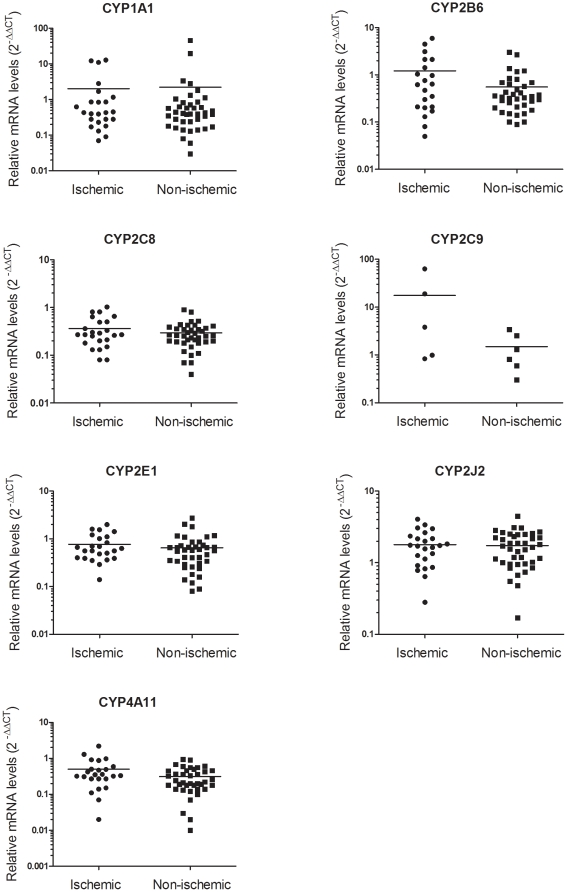
CYP450 mRNA levels in ischemic and non-ischemic heart samples. Comparison of the relative levels of CYP450 mRNAs in samples from hearts with a diagnosed ischemic status (n = 25) or non-ischemic status (n = 41). No major differences were observed in the relative expression of CYP450 mRNAs under these conditions. Levels were determined and compared using the 2^−ΔΔCT^ method with a calibrator.

We compared CYP450 mRNA levels in ischemic (n = 24) and non-ischemic (n = 40) hearts from patients with end-stage heart failure. Figure 2 indicates that expression levels were unaffected by ischemic status. However, CYP2C9 mRNA levels tended to be higher in ventricular samples from ischemic hearts than from non-ischemic ventricles.

#### Comparison of the relative cardiac CYP450 mRNA levels between male and female patients

No differences in mRNA levels were observed between males and females except for CYP2J2, for which mRNA levels were lower in men compared to women (1.5±0.8 *vs.* 2.2±0.8; p<0.01).

#### Comparison of the relative cardiac CYP450 mRNAs between left and right ventricular samples

**Figure 3:**
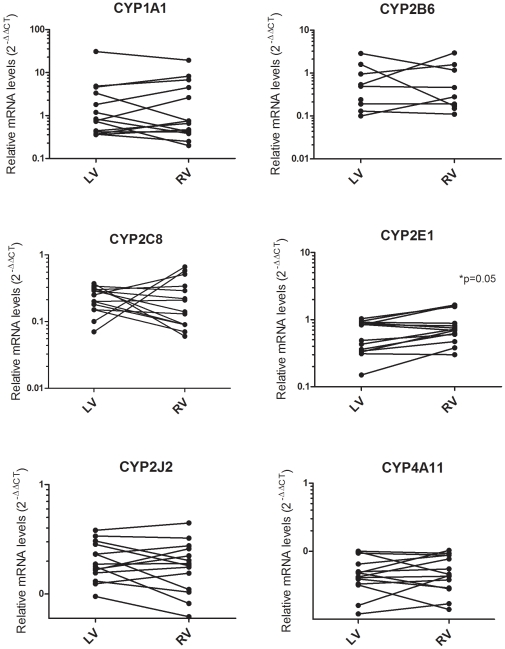
CYP450 mRNA levels in left and right ventricular heart samples. Comparison of the relative levels of CYP450 mRNAs in samples from paired left and right ventricles (n = 7). No major differences were observed in the relative expression of CYP450s in the left and right ventricular tissues except for CYP2E1 mRNAs, where higher levels tended to be observed for the right ventricle (1.0±0.5 *vs* 0.6±0.3; p = 0.05). Levels were determined and compared using the 2^−ΔΔCT^ method with a calibrator. mRNA from CYP2C9 was detected in one heart (data not shown). RV: right ventricle; LV: left ventricle.

Samples were obtained from a limited number of subjects (n = 7) from both their left and right ventricles, thereby making it possible to compare these two tissue sites (Figure 3). Overall, no major differences were observed in the relative expression of CYP450s except for CYP2E1, for which higher levels tended to be observed in the right ventricles (1.0±0.5 *vs.* 0.6±0.3; p = 0.05).

### Detection of CYP450 proteins in human cardiac myocytes

**Figure 4:**
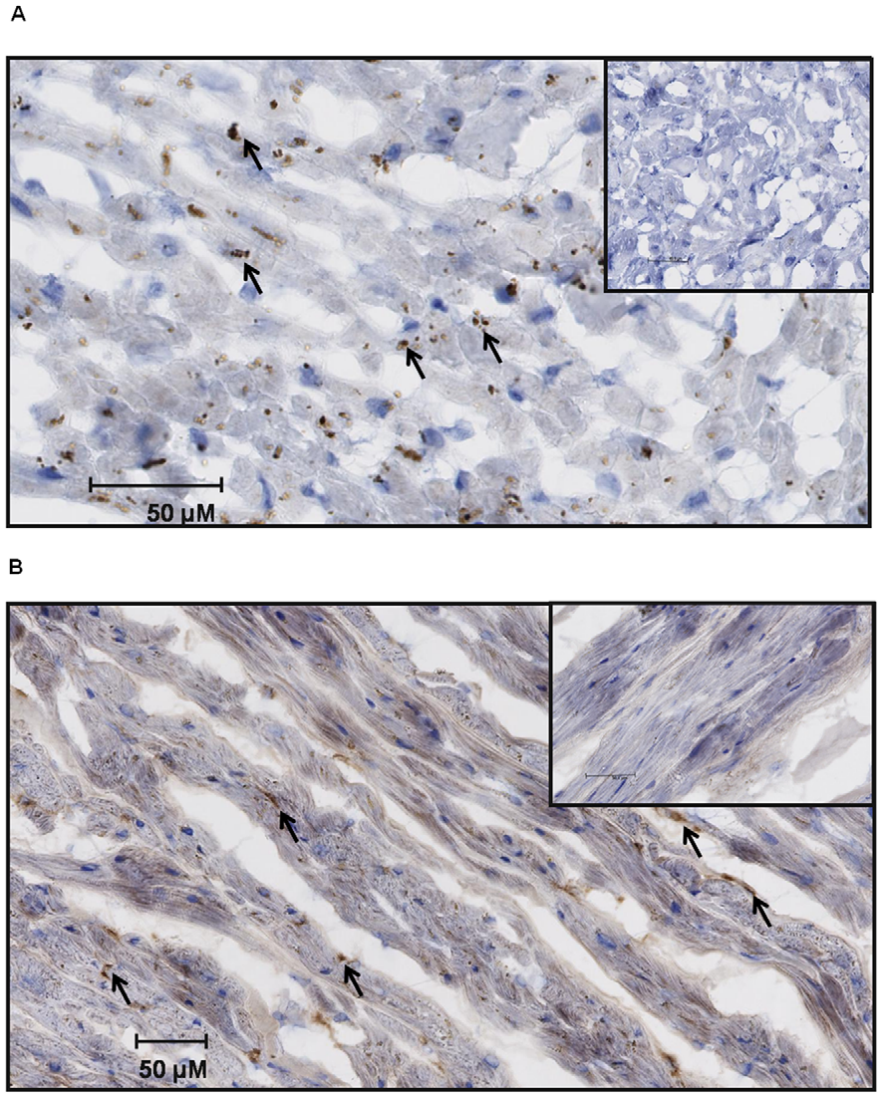
CYP2C and CYP2J2 immunohistochemical staining in human hearts. Immunohistochemical staining of human heart samples. The presence of CYP2Cs (upper panel) and CYP2J2 (lower panel) was revealed by dark elements inside the cardiomyocytes, which are absent in negative controls (inserts).

Immunohistochemical studies were performed to confirm CYP450 expression in human cardiomyocytes. Primary antibody negative controls and CYP450 antibody positive staining results clearly indicated that CYP2J2 and CYP2Cs proteins were expressed in human cardiomyocytes (Figure 4). It should be noted that our immunohistochemical studies included a control for potential false positive results with lipofuscine.

### CYP450 activities in human heart microsomes

The method described here was developed and validated for the preparation of human heart microsomes that retain metabolic activities. More than 40 combinations of buffer, co-factors and antioxidant agents were tested, including TRIS *vs.* phosphate buffer at a range of pH values, sucrose, glucose, glycerol, detergents (CHAPS, TRITION), and Complete®. The optimal mixture based on maximal metabolic activity was obtained with a phosphate buffer containing potassium chloride, PMSF and DTT combined with sonication procedures as described in the Methods section.

#### Verapamil metabolism in human heart microsomes

**Figure 5:**
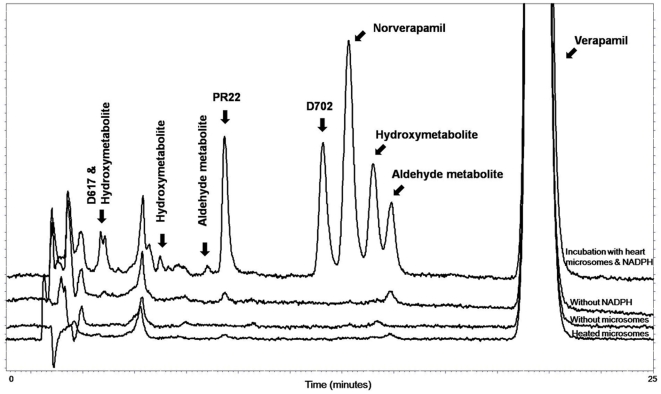
Verapamil and its metabolites produced following incubation with human heart microsomes. HPLC chromatogram depicting nine metabolites detected following the incubation of verapamil with microsomes prepared from human heart ventricles. Structure allocation was established following peak collection and reanalysis by LC-MSMS according to Walles et al. assay.[26] Metabolites PR22, D702, norverapamil and a hydroxymetabolite (retention time 14 min) were present in sufficient amounts to allow quantification. The characterization of CYP450-dependency on the formation of four major verapamil metabolites by microsomes prepared from human heart samples is illustrated. Metabolites could not be detected in the absence of a NAPDH regenerating system or in the presence of pre-heated microsomes.

Incubations were performed using the probe-drug substrate verapamil. Figure 5 shows that 9 verapamil metabolites could be detected and 4 metabolites quantified following 4-hour incubations with human heart microsomes prepared according to our procedure. We demonstrated that the formation of verapamil metabolites was time-dependent and linearity maintained for the 4-hour incubation period. The formation rate of verapamil metabolites was concentration dependent (50, 100 and 400 µM of verapamil): the amount of metabolites formed increased 2-4 fold in this range of concentrations. Verapamil metabolism was completely eliminated in the absence of a functional NADPH regenerating-system (Figure 5), thereby confirming that metabolite formation was not due to chemical degradation of the substrate. As well, metabolite formation was almost completely eliminated in incubations with CO or nitrogen gas.

**Figure 6:**
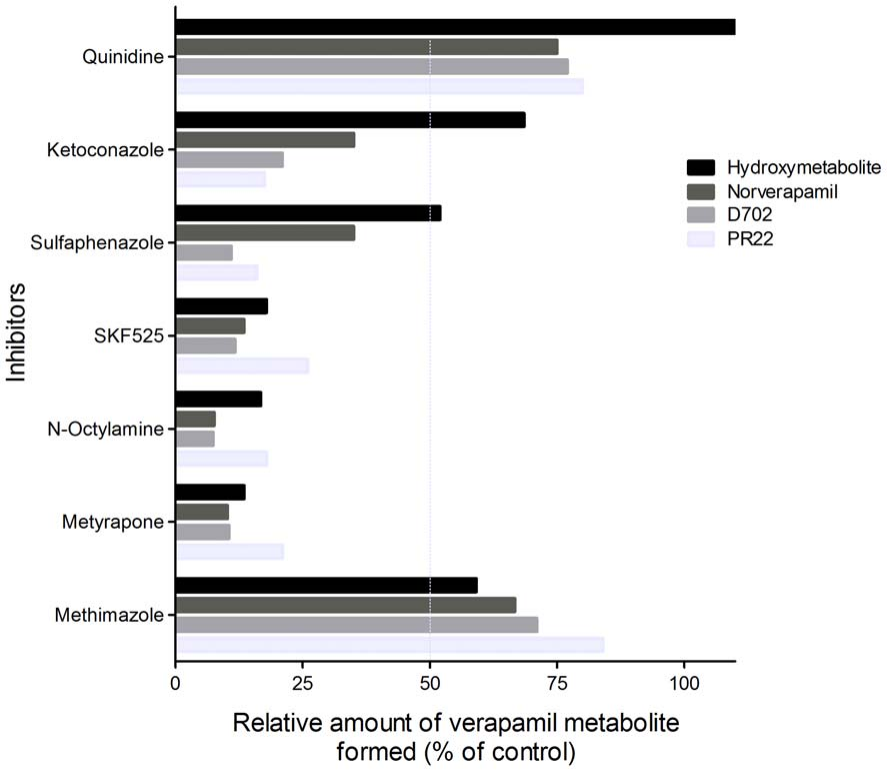
Effects of CYP450 chemical inhibitors. Inhibition of the formation of the four major verapamil metabolites by chemical inhibitors of CYP450s. Significant inhibition in the formation of most metabolites was observed with potent CYP450 inhibitors (SKF525, n-octylamine, metyrapone) while slight inhibition was observed with the FMO inhibitor methimazole.

The effects of chemical inhibitors on the metabolism of verapamil were studied using metyrapone, N-octylamine, SKF-525 and methimazole. Results of these studies are presented in Figure 6. When potent CYP450 inhibitors (metyrapone, N-octylamine or SKF525) were added to the incubation mixture, the formation of all major verapamil metabolites decreased significantly (more than 80%). In contrast, methimazole (a strong inhibitor of flavoprotein monooxygenase (FMO) enzymatic system) only slightly decreased the formation of verapamil metabolites. To further discriminate between the contribution of FMOs and CYP450s, the metabolism of verapamil was evaluated with human heart microsomes previously heated at 60°C for 10 minutes. This procedure is known to eliminate CYP450 activity while not affecting FMOs. Results showed that pre-treatment of human heart microsomes by heating blunted metabolic activity (Figure 5). These results support the claim that the CYP450 enzymatic system contributes to the metabolism of verapamil in human heart microsomes.

Additional experiments were performed with selective chemical inhibitors for various CYP450 isozymes. A significant decrease (30–90%) was observed in the formation of various metabolites following incubation of verapamil with sulfaphenazole (10 µM), a CYP2C inhibitor, or ketoconazole (1 µM), a compound known to inhibit CYP3As and CYP2J2. In contrast, no significant inhibition was observed even with high concentrations (5 µM) of quinidine (CYP2D6 inhibitor) (Figure 6).

#### Inter-subject variability in CYP450 activities from human heart microsomes

**Figure 7:**
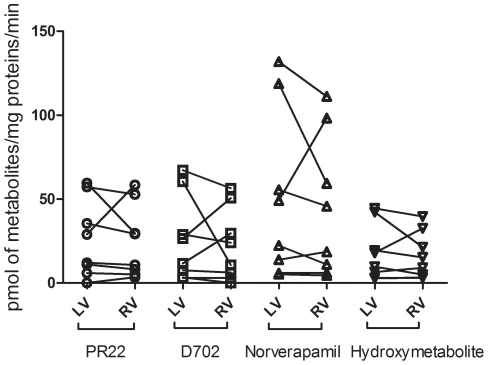
Metabolism of verapamil in right versus left ventricles. Comparison of the formation of four major verapamil metabolites by microsomes prepared from left and right paired ventricles of human heart samples (n = 8).

A wide variability in the profile of verapamil metabolism was observed among microsomes prepared from various human heart samples (n = 20). Median values for the formation rate of verapamil metabolites produced by left ventricular microsomes (range; minimum-maximum values) were 27 (2.1–76), 21 (3.2–108), 25 (5.8–214) and 12 (3.2–81) pmol/min/mg of heart microsomal proteins for PR22, D702, norverapamil and the hydroxymetabolite, respectively. In microsomes prepared from right ventricles (n = 8), median values were 21 (3.7–64.4), 21 (3.2–74), 39 (5.8–145) and 16 (3.0–48) pmol/min/mg of heart microsomal proteins for PR22, D702, norverapamil and the hydroxymetabolite, respectively. Figure 7 gives the values measured for each metabolite in hearts (n = 8) for which microsomes were prepared from both left and right ventricles. Data obtained suggest that both tissues display similar activity levels (p>0.4).

#### Enantioselective metabolism

**Figure 8:**
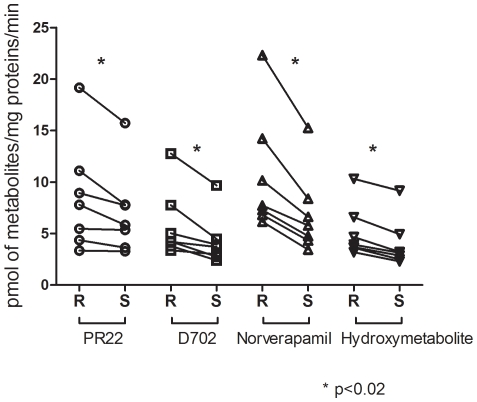
Stereoselectvity of verapamil metabolism in human hearts. Stereoselective metabolism in the formation of the four major verapamil metabolites by microsomes from human heart ventricles (n = 7).

We performed incubations with human heart microsomes using either R-verapamil or S-verapamil as substrates. In all human heart microsomes tested (n = 7) and for all metabolites, the formation rates measured for the R-enantiomer were higher (p<0.02) than those measured with the S-enantiomer (Figure 8).

## Discussion

Results obtained in this study are derived from the largest cohort of human heart samples (n = 75) ever analyzed for determining the relative mRNA levels for CYP450 isozymes involved in drug-metabolism. We confirmed that CYP2J2 mRNAs were the most abundant in the left and right ventricles of human hearts with an increased expression in females compared to males. Our results also indicated that CYP450 mRNA levels were not affected by ischemic or non-ischemic conditions in patients with end-stage heart failure and suggested that CYP450 mRNAs were present at similar levels in both the left and right ventricles. Lastly, we noticed that CYP2C9 was detected in less than 10% of human hearts. We also determined CYP450 activity in human heart microsomes using verapamil as a probe-drug substrate by showing the formation of nine metabolites. In contrast to the stereoselectivity observed in the liver for the metabolism of verapamil preferred metabolism of the R-enantiomer was demonstrated. It should be noted that our results were obtained with hearts from patients with end-stage heart failure and that had previously been exposed to many drugs.

Using immunoblotting techniques, Zeldin's group reported in 1996 that CYP2J2 was highly expressed in human heart tissues.[11] Their study was followed by Thum and Borlak's study using RT-PCR, which suggested that several CYP450s were expressed in the human heart.[10] Drawing on data obtained from 7 explanted hearts from heart failure patients and 2 hearts from normal subjects, they reported that the presence of CYP1A1, CYP2B6, CYP2C8, CYP2C19, CYP2D6 and CYP4B1 mRNAs. No mRNA for CYP3A4, CYP3A5 or CYP3A7 could be detected.[10] Using a semi-quantitative assay and a pool of human heart samples (n = 3), they further demonstrated that gene expression for CYP2J2 and CYP1A1 was the most abundant.[26] They confirmed these results in 11 diseased and 2 normal human hearts, showing that CYP450 gene expression in the left ventricle was limited to CYP1A1, CYP2A6, CYP2B6, CYP2C8, CYP2E1, CYP2J2 and CYP4A11.[21] Recent studies conducted with quantitative real-time PCR and samples from 8 explanted human hearts indicated that CYP2J2 mRNA values were >900 times higher than those measured for CYP2C9 or CYP2C8.[12] Our results, obtained in a large cohort of human hearts, support data from these pilot studies and confirm high CYP2J2 mRNA levels in human hearts. Furthermore, our access to a large number of human hearts enabled us to demonstrate high inter-subject variability, especially for CYP2J2 and CYP1A1 mRNA levels.

It is noteworthy that CYP2C9 and CYP2B6 mRNAs were only detected in a small number of hearts. However, CYP2C9 mRNA levels tended to be greater in samples from ischemic hearts. This result supports data obtained in rats indicating that chronic hypoxia led to an increase in rat CYP2C9 expression in mesenteric arteries.[27] It has also been reported that hypoxia increases the expression of CYP2C mRNAs and proteins and enhanced 11,12-EET production in human endothelial cells.[28] The fact that CYP2C9 mRNA was detected in only 10% of our samples and increased in ischemic hearts may suggest that a certain amount of this mRNA originates from endothelial cells in some but not in other ventricular samples.

We observed that CYP2J2 mRNA levels were higher in female than in male left ventricular samples. One could suggest that the sex-dependant CYP2J2 mRNA expression explains higher susceptibility of men for the onset or progression of cardiovascular diseases. This hypothesis needs to be investigated and confirmed by other studies. Gender-differences in the expression of CYP450s isozymes are also an interesting observation. Earlier studies have reported that CYP3A4 expression (mRNA and protein levels) is sex-dependant expression, with higher activity measured in women.[29], [30], [31]

Thum and Borlak's group suggest that CYP450 mRNAs are predominantly expressed in the right ventricle.[10] In contrast, our results clearly show the presence of CYP450 mRNAs in the left ventricles. The only exception could be for CYP2E1. A similar extent of metabolism measured in the formation of verapamil metabolites from both the left and right ventricles further confirms our observations.

Results obtained by immunohistochemistry analyses demonstrate that CYP450 proteins such as CYP2J2 and CYP2Cs are expressed in human cardiomyocytes. These results are in line with observations by DeLoziers et al., although they did not control for false positive results due to lipofuscine.[12] In our study, the presence of CYP450 proteins was clearly demonstrated inside cardiomyocytes away from nucleus and detritus residues of proteins accumulated by lipofuscine.

Our final series of experiments aimed at demonstrating CYP450 activities in microsomes prepared from human heart samples. This objective required the development and validation of standardized procedures for the reproducible preparation of microsomes that retain activity. After several attempts, microsomes from 20 human heart samples were prepared and used to characterize the metabolism of the probe-drug verapamil. Nine metabolites were detected following incubations with verapamil. We demonstrated that the formation of these metabolites was CYP450 dependent and unrelated to FMO activity. We also demonstrated that left and right ventricles possess similar CYP450 activity levels. Inconsistent results had been previously reported in small studies for verapamil and testosterone.[10], [21], [26] Our study is the first to report extensive and reproducible data pertaining to the metabolism of drug in human cardiac tissue.

Verapamil is a calcium channel blocking agent commonly prescribed in the treatment of angina pectoris, coronary artery disease, cardiac arrhythmias and hypertension. Verapamil undergoes extensive hepatic first-pass metabolism; O-demethylation, N-demethylation and N-dealkylation are the major verapamil metabolism pathways. Previous studies have shown that CYP3A4, CYP3A5, CYP2C8 and, to a much lesser extent, CYP2E1 and CYP1A2 are involved in the metabolism of verapamil.[32], [33], [34], [35] Verapamil is used clinically as a racemic mixture of S- and R-enantiomers having various pharmacokinetic and pharmacological properties. Following oral administration, the S-enantiomer is preferentially metabolized upon first-pass metabolism, and plasma levels are lower than those of the R-enantiomer.[36] The S-enantiomer is also the eutomer for calcium channel blocking.[37] We report for the first time on the stereoselective metabolism of verapamil by human heart microsomes. The same metabolism pattern (i.e., the same metabolites appeared to be formed) was observed for both verapamil enantiomers. However, the formation rates of all metabolites were higher for R-verapamil than for the S-enantiomer (p<0.02). This finding underlines the importance of studying CYP450 activity in tissues expressing the effector protein to gain a better understanding of drug action.

In conclusion, we performed a thorough determination of CYP450 mRNA levels using the largest cohort of human hearts ever analysed. This study pertains to the role of cardiac CYP450s in cardiac-specific drug metabolism and, by extension, to cardiac pathophysiology. To date, little is known about site-specific drug-drug interactions as they relate specifically to the heart. In particular, while liver microsomes express low levels of CYP2J2 and are therefore not taken into account in routine drug-drug interaction screening, CYP2J2 is highly expressed in the heart and could be extremely relevant for the local clearance of drugs and metabolite formation in the heart.

## Materials and Methods

Approval for the use of human tissue materials was obtained from the Ethics Review Board at the Montreal Heart Institute and fully complied with procedures from the Réseau d'Échange de Tissus et Échantillons Biologiques du Québec (RETEB). Written consent was obtained from all patients from whom tissues were obtained.

### Preparation of samples

Heart samples were obtained from patients suffering from end-stage heart failure and undergoing heart transplant. Immediately after explantation, the hearts were immersed in a cold transplantation buffer, rapidly mounted on an extracorporal circulating pump and perfused for 10 minutes with the same buffer. Left and right ventricular samples (4–10 g) were prepared from the apex region outside of apparent ischemic scars and immediately frozen in liquid nitrogen. RNA was isolated and microsomes prepared in thawed samples after removal of fat, fibrous tissue, and large vessels apparent in the samples.

### CYP450 mRNA levels in human hearts

#### Isolation of RNA and preparation of cDNA

Total RNA was extracted from 68 human explanted hearts from patients with end-stage heart failure (n = 68). About 50 mg of left ventricular tissue was homogenized in 1 mL of Trizol and transferred to a 2 mL tube. Seven paired samples available from the right ventricles of patients were processed in the same way. Chloroform (200 µL) was added, the mixture shaken for 15 seconds, and then centrifuged at 12,000 g for 15 minutes. The upper aqueous layer was transferred to a new tube and ethanol 70% was added (1∶1 volume). RNA was extracted using the Qiagen kit (RNeasy Mini kit; Qiagen Sciences, MD, USA) according to the manufacturer's recommendations. RNA quality was assessed by determining the variability of CT values of GAPDH since this marker as been identified as a valid housekeeping gene in heart tissues. Total RNA (1 µg) from each sample was used for reverse transcription. RNA, random primers (3 µg) and dNTP (25 µM) were preheated for 5 minutes at 65°C, then 5X-first strand buffer, 40 units of RNAse inhibitor, DTT (0.01M) and 200 units of Superscript II (Invitrogen, Carlsband, CA, USA) and diethylpyrocarbonate-treated water were added to a final volume of 20 µL. Reverse transcription was carried out for 50 minutes at 42°C and stopped by heating to 70°C for 15 minutes. The resulting cDNA was frozen at −20°C until analyzed.

#### Real-time PCR analysis

Quantitative real-time PCR was performed using TaqMan probe and primer sets from Applied Biosystem (Foster, CA, USA). The assay IDs were: CYP1A1 (Hs00153120_m1), CYP2B6 (Hs0059368_m1), CYP2C8 (Hs00946140_g1), CYP2C9 (Hs00426397_m1), CYP2E1 (Hs0059368_m1), CYP2J2 (Hs00356035_m1), CYP4A11 (Hs00167961_m1) and GAPDH (Hu_GAPDH). cDNA was diluted 20-fold (10 ng/reaction), mixed with TaqMan PCR Master Mix and amplified using cycling conditions for 50 cycles. Reactions were run in a RotorGene Detector model 6000 (Corbett Research, Mortlake, Australia).

Comparison of the relative mRNA expression of various CYP450s was performed using the ΔCT method (CT_CYP450_-CT_GAPDH_) to ascertain relative levels of isoenzymes among them. Data were expressed as the ratio of the target mRNA to GAPDH mRNA (2^−ΔCT^).[38], [39] For their part, mRNA levels associated with the expression of each isozyme within a specific condition (ischemic *vs.* non-ischemic heart samples, male *vs.* female and left *vs.* right ventricles) were determined using a calibrator and the 2^−ΔΔCT^ method.[38] Determination of mRNA levels was performed in triplicate for each gene, and two independent experiments were repeated to confirm results.

### Detection of CYP450 proteins in human cardiomyocytes by immunohistochemistry

Immunohistochemical detection was performed on human frozen tissues using a Discovery XT system (Ventana Medical Systems, Tucson, AZ). After antigen retrieval with proprietary reagents, primary antibodies anti-CYP2J2 (sc-66364) and an anti-Cytochrome P450 clone 2C8+2C9+2C19+2C12 (ab22596) were applied. Goat anti-human CYP2J2 (Santa Cruz Biotechnology, Inc., CA) was incubated for 2 hours at room temperature. Rabbit anti-human Cytochrome P450 clone 2C8+2C9+2C19+2C12 (Abcam Inc, Cambridge, MA) was incubated for 60 minutes at room temperature. Sections were then incubated with a secondary biotinylated anti-goat or anti-rabbit antibody (Jackson ImmunoResearch). Streptavidin horseradish peroxidase and 3,3′-diaminobenzidine were used according to the manufacturer's instructions (Ventana Medical Systems). Lastly, sections were counterstained with hematoxylin and analyzed by standard light microscopy.

### CYP450 activities in human heart microsomes

#### Materials

Verapamil, S-verapamil, R-verapamil and norverapamil were purchased from Sigma-Aldrich (St. Louis, MO, USA). Metyrapone, methimazole, SKF525A, N-octylamine and cofactors (NADP+, D-glucose 6-phosphate and glucose 6-phosphate dehydrogenase) were also obtained from Sigma-Aldrich (St. Louis, MO, USA). EDTA was purchased from JT Baker (Phillipsburg, NJ, USA). Other chemicals used were of the highest quality commercially available.

#### HPLC analysis

The HPLC system (Thermo Separation products, Fremont, CA) consisted of a SpectraSystem P4000 pump, a SpectraSystem AS3000 autosampler, a FL3000 fluorescence detector, a SpectraSystem UV3000 ultraviolet detector and PC1000 System Software. Quantification of verapamil and major verapamil metabolites was performed after slight adjustments to the assay described by Wang *et al*.[40] Briefly, separation of verapamil metabolites was achieved on a phenyl-hexyl column (5 µM ×250 mm ×4.6 mm; Phenomenex, CA, USA) using a mobile phase containing a potassium phosphate buffer (10 mM, pH 7.0), acetonitrile, methanol and trietylamine (40∶38∶22∶0.1). Eluent was monitored by fluorescence absorbance at λ = 204 and 314 nm (absorption and emission wavelengths, respectively). Intraday and interday coefficients of variation were less than 5% and 10%, respectively.

#### Preparation of human heart microsomes

Frozen samples of human heart ventricles (4–10 g) were immersed in cold phosphate buffer 100 mM (pH 7.4) containing potassium chloride 150 mM, EDTA 1 mM, dithiotreitol 0.5 mM and PMSF 0.01 mM (1∶3, w/v). Tissue was roughly sliced prior to homogenization using a Polytron. The resulting suspension was sonicated 3 times for 5–10 seconds. Homogenization and sonication were performed on ice. Microsomal fractions were isolated by centrifugation at 10,000 g for 20 minutes. The resulting supernatant was centrifuged twice at 100,000 g for 2 consecutive 90-minute periods. The final microsomal pellet was resuspended in a phosphate buffer 100 mM (pH 7.4), potassium chloride 150 mM and EDTA 1 mM. All procedures were performed at 4°C. Incubations with verapamil were performed on freshly isolated microsomes. The leftover heart microsomes were immediately shock frozen in liquid nitrogen for subsequent protein quantification. Microsomal heart proteins were quantified using a BCA Pierce kit.

#### In vitro incubations

All microsomal incubations were performed in duplicate. The standard incubation mixture (final volume, 500 µL) consisted of microsomes (200 µL), 195 µL phosphate buffer 50 mM (pH 7.4), 100 µL NADPH-regenerating system solution and 5 µL substrate (verapamil 50, 100 or 400 µM). The NADPH-regenerating solution contained NADP^+^ (498 µg), D-glucose 6-phosphate (502 µg), MgCl_2_ 5 mM and 0.2 U glucose 6-phosphate dehydrogenase.

Incubations containing microsomes, buffer and NADPH-regenerating system solution were pre-incubated at 37°C for 10 minutes. Reactions were initiated by the addition of the substrate (verapamil) to the incubation mixture. The enzymatic process was stopped by adding 500 µL of ice-cold acetonitrile. Different incubation times were assessed: 15 minutes to 4 hours. Incubation mixtures were centrifuged at 15,000 rpm for 10 minutes. Supernatants (30 µL) were injected into the HPLC system and the major metabolites of verapamil were monitored.

#### Inhibition studies

Different studies were conducted to evaluate the potential contribution of CYP450s on the metabolism of verapamil by human heart microsomes. Drug inhibitors (metyrapone 100 µM, SKF525 100 µM, N-octylamine 100 µM or methimazole 200 µM) were added to incubation mixtures containing human heart microsomes. Isozyme selective inhibitors, sulfaphenazole (10 µM), ketoconazole (1 µM) and quinidine (5 µM) were also evaluated. Briefly, the reaction mixtures containing enzymatic sources, potassium buffer, NADPH-regenerating system and inhibitor were warmed at 37°C for 10 minutes prior to the addition of verapamil (400 µM). Formation rates of verapamil metabolites were expressed as a percentage of metabolites formed relative to the amounts of the same metabolites formed in incubations containing no inhibitor.

A series of incubations was also performed with previously inactivated human heart microsomes. The CYP450 proteins were inactivated by heating microsomes at 60°C for 10 minutes. Incubations were also conducted in the presence of nitrogen gas or carbon monoxide gas in replacement of ambient air to inactivate CYP450s. The extent of verapamil metabolism was measured as a percentage of control activity.

#### NADPH-dependent enzymatic system

Verapamil metabolite formation was studied in incubations performed with or without a NADPH-regenerating system (incubations without NADPH contained only vehicle, ie MgCL_2_).

### Statistical analysis

Data are expressed as mean ±S.D. and when possible, individual data are presented. Relative mRNA levels for various isozymes were compared using a two-way ANOVA. The comparison of CYP450 mRNA expression levels in failing hearts with ischemic or non-ischemic disease was performed using an unpaired t-test with Welch's correction. An unpaired t-test with Welch's correction was also used assess and compare male and female mRNA levels. mRNA levels for each isozyme in the left and the right ventricles for each patient were compared using a Wilcoxon signed rank test. This test was used when formation rate of verapamil metabolites by left and right ventricles was studied (paired samples of left and right ventricles from the same patients). The metabolism of S-verapamil and R-verapamil within human heart microsomes was also compared using the Wilcoxon signed rank test. Differences were considered significant at p value <0.05.
